# The inositol pyrophosphate metabolism of *Dictyostelium discoideum* does not regulate inorganic polyphosphate (polyP) synthesis

**DOI:** 10.1016/j.jbior.2021.100835

**Published:** 2022-01

**Authors:** Yann Desfougères, Paloma Portela-Torres, Danye Qiu, Thomas M. Livermore, Robert K. Harmel, Filipy Borghi, Henning J. Jessen, Dorothea Fiedler, Adolfo Saiardi

**Affiliations:** a Medical Research Council, Laboratory for Molecular Cell Biology, University College London, London, WC1E 6BT, UK; b Institute of Organic Chemistry, CIBSS - The Center for Integrative Biological Signalling Studies, University of Freiburg, Albertstr. 21, 79104, Freiburg, Germany; c Leibniz-Forschungsinstitut für Molekulare Pharmakologie, Robert-Rössle-Straße 10, 13125, Berlin, Germany

**Keywords:** Metabolism, ip6k, ppip5k, Inorganic polyphosphates, Inositol, Phosphate, Amoeba, Development

## Abstract

Initial studies on the inositol phosphates metabolism were enabled by the social amoeba *Dictyostelium discoideum.* The abundant amount of inositol hexakisphosphate (IP_6_ also known as Phytic acid) present in the amoeba allowed the discovery of the more polar inositol pyrophosphates, IP_7_ and IP_8_, possessing one or two high energy phosphoanhydride bonds, respectively. Considering the contemporary growing interest in inositol pyrophosphates, it is surprising that in recent years *D. discoideum*, has contributed little to our understanding of their metabolism and function. This work fulfils this lacuna, by analysing the *ip6k, ppip5k* and *ip6k-ppip5K* amoeba null strains using PAGE, ^13^C-NMR and CE-MS analysis. Our study reveals an inositol pyrophosphate metabolism more complex than previously thought. The amoeba Ip6k synthesizes the 4/6-IP_7_ in contrast to the 5-IP_7_ isomer synthesized by the mammalian homologue. The amoeba Ppip5k synthesizes the same 1/3-IP_7_ as the mammalian enzyme. In *D. discoideum*, the *ip6k* strain possesses residual amounts of IP_7_. The residual IP_7_ is also present in the *ip6k-ppip5K* strain, while the *ppip5k* single mutant shows a decrease in both IP_7_ and IP_8_ levels. This phenotype is in contrast to the increase in IP_7_ observable in the yeast *vip1*Δ strain. The presence of IP_8_ in *ppip5k* and the presence of IP_7_ in *ip6k-ppip5K* indicate the existence of an additional inositol pyrophosphate synthesizing enzyme. Additionally, we investigated the existence of a metabolic relationship between inositol pyrophosphate synthesis and inorganic polyphosphate (polyP) metabolism as observed in yeast. These studies reveal that contrary to the yeast, Ip6k and Ppip5k do not control polyP cellular level in amoeba.

## 1 Introduction

The social amoeba *Dictyostelium discoideum* was one of the primary experimental models to study inositol phosphate metabolism and signalling in the 1980s (Europe-Finner et al., 1991). The lipid independent route to IP_6_ synthesis was identified (Stephens and Irvine, 1990) in *D. discoideum,* and inositol species more polar than the fully phosphorylated ring of IP_6_, the inositol pyrophosphate (see below) were also discovered in this organism (Stephens et al., 1993). By the middle of the 90s, it was discovered that phospholipase C in *D. discoideum* is not required to produce IP_3_ nor to control calcium signalling (Van Dijken et al., 1995, 1997). Perhaps for these reasons, the interest of inositol scientists in this organism faded over the years. However, the interest in *D. discoideum* did not completely disappear. At the start of the new millennium, the social amoeba was used to study the effect on the inositol phosphate metabolism of lithium and other mood stabilizing drugs (King et al., 2010; Williams et al., 1999, 2002). More recently, *D. discoideum* was used to characterise the roles of inositol polyphosphate in programmed cell death (Al-Anbaky et al., 2018) and to characterise the phytocannabinoid-dependent mTORC1 regulation by the inositol polyphosphate multikinase (Damstra-Oddy et al., 2021). However, the precise description of the inositol phosphate metabolic pathway and the characterization of the different inositol kinase mutants is still missing in the amoeba. To our knowledge, only the IP6K (Ip6K also referred to as I6kA) null strain (*ip6k, i6kA*) has previously been generated. The previously characterized *ip6k* amoeba (Luo et al., 2003) possess a biochemical phenotype, the absence of inositol pyrophosphate, similar to the mutant of the homologous yeast *Saccharomyces cerevisiae* gene Kcs1 (*kcs1*Δ) (Saiardi et al., 2000).

The IP6K, as the name indicates, phosphorylates IP_6_ to generate the inositol pyrophosphate IP_7_ (Saiardi et al., 1999). There is at least one other class of kinases able to synthesize inositol pyrophosphate known as PPIP5K (*S. cerevisiae* Vip1; *Schizosaccharomyces pombe* Asp1) that, as the name suggests, primarily phosphorylates IP_7_, also abbreviated to PP-IP_5_, IP_8_ or (PP)_2_-IP_4_ (Choi et al., 2007; Fridy et al., 2007; Mulugu et al., 2007).

The yeast and mammalian IP6K and PPIP5K have been extensively characterized. The mammalian IP6K is able to pyro-phosphorylate position five of the inositol ring generating the isomer 5PP-IP_5_ (hereafter 5-IP_7_) (Draskovic et al., 2008), while mammalian PPIP5K phosphorylate position one physiologically generating 1,5(PP)_2_-IP_4_ (hereafter 1,5-IP_8_) (Lin et al., 2009; Wang et al., 2012).

*D. discoideum* possesses one Ip6k gene and a Ppip5k homologous gene (see below). The similar enzymology between human, yeast and amoeba suggests a similar inositol pyrophosphate metabolism. However, this appears not to be the case since ^1^H,^31^P-NMR spectroscopy and enzymology studies (Laussmann et al., 1996, 1997) suggest that the amoeba possesses a different form of IP_8_, the isomer 5,6(PP)_2_-IP_4_ (hereafter 5,6-IP_8_).

Inositol pyrophosphates are attracting a growing interest due to their link to metabolic disorders including obesity and diabetes (Mukherjee et al., 2020), human diseases, such as cancer and Alzheimer's (Crocco et al., 2016), combined with improved tools to facilitate their analysis *in vivo* (Harmel et al., 2019; Ito et al., 2018; Qiu et al., 2020; Wilson et al., 2015). The picture that is emerging is that inositol pyrophosphates regulate basic energy metabolism (Szijgyarto et al., 2011) through their ability to control phosphate homeostasis (Azevedo and Saiardi, 2017; Saiardi, 2012). The discovery that in *S. cerevisiae,* the IP_7_ synthesizing enzyme Kcs1 regulates the metabolism of polymeric linear chains of phosphate groups, also known as inorganic polyphosphate (polyP) (Lonetti et al., 2011), led to the discovery of a novel signalling paradigm involving the SPX protein domain (Wild et al., 2016). In this case, the interaction between IP_7_ and the SPX domain of the *S. cerevisiae* polyP-synthesizing enzyme, Vtc4, stimulates polyP synthesis (Gerasimaite et al., 2017). The SPX domain is present in dozens of plant proteins involved in phosphate homeostatic regulation (Azevedo and Saiardi, 2017; Secco et al., 2012). Due to the limited availability and the socioeconomic importance of phosphate as fertiliser, the roles played by inositol pyrophosphate in regulating plant phosphate absorption and metabolism is now one area of intense investigation (Dong et al., 2019; Riemer et al., 2021; Zhu et al., 2019). However, recent work carried out in *S. pombe*, indicates that it is not Kcs1 but the PPIP5K homologous enzyme, Asp1/Vip1, which regulates Vtc4-driven polyP synthesis in fission yeast (Pascual-Ortiz et al., 2021). Therefore, our understanding of the exact roles played by inositol pyrophosphates in polyP synthesis and phosphate homehostasis is far from complete.

Nevertheless, the absence of polyP in *S. cerevisiae kcs1*Δ has been highly influential. In fact, *D. discoideum ip6k* knockout has been utilized as a proxy, using unrefined supporting data, which demonstrates that the amoeba has low polyP level (Suess and Gomer, 2016). Amoeba synthesize polyP through the polyphosphate kinase (Ppk1), a gene acquired from bacteria by horizontal gene transfer (Livermore et al., 2016). We discovered that polyP hugely accumulates during the *D. discoideum* developmental program (Livermore et al., 2016). The polyP-induced synthesis following the starvation/aggregation signal leads to its secretion (Suess and Gomer, 2016).

Like inositol pyrophosphates, polyP is also attracting a growing interest. PolyP, has been described as an important primordial chaperone able to regulate the aggregation of proteins which form in neurodegenerative disorders (Cremers et al., 2016). However, it is also important to mitochondrial physiology (Solesio et al., 2021) driving a novel protein post translational modification, lysine-polyphosphorylation (Azevedo et al., 2015); and controlling several aspects of the blood coagulation cascade (Morrissey et al., 2012). Since the many important roles attributed to polyP, it is of fundamental importance to understand if the link between inositol pyrophosphates (either IP_7_ or IP_8_) and polyP synthesis is an evolutionarily conserved feature.

*D. discoideum* offers the unique opportunity to address these issues since it is an excellent experimental model for both inositol pyrophosphate and polyP studies (Desfougeres and Saiardi, 2020). Here we characterise the amoeba inositol pyrophosphate metabolic pathway by creating the *ip6k*, the *ppip5k* and the double *ip6k-ppip6k* strain in the AX2 genetic background to verify if these enzymes are regulating polyP synthesis.

## 2 Materials and methods

### 2.1 Identification *D. discoideum* inositol phosphate kinase genes

To identify the amoeba inositol phosphate genes, we performed Protein Basic Local Alignment Search Tool (BLAST) searches using all inositol phosphate kinases found in *S. cerevisiae* and *H. sapiens* against the *D. discoideum* complete genome as previous described (Laha et al., 2021).

### 2.2 Genetic manipulations

Yeast transformations were performed using the lithium/acetate method (Gietz and Woods, 2002). Yeast knockouts were generated using well-described procedures (Janke et al., 2004). The correct removal of the genes were first verify by PCR and then phenotypically characterising the inositol phosphate profile by ^3^H-inositol-labeling Sax-HPLC analysis (Azevedo and Saiardi, 2006).

### 2.3 Cloning *D. discoideum* Ppip5K in yeast expression vector

Codon-optimisation of *D. discoideum* Ppip5K sequences for yeast expression was designed through an interface from SciTools® (Integrated DNA Technology). Restriction sites were added at the 5′ *Sal*I and 3’ NotI to cloned Ppip5K in a pADH-GST plasmid (Azevedo et al., 2015).

### 2.4 Growth of yeast and amoeba

Yeast were grown in rich (YPD: 1% yeast extract, 2% peptone, 2% dextrose) or synthetic complete medium (SC, Formedium) in the absence or presence of uracil to select for auxotrophy. For [^3^H]-inositol labelling, the cells were grown in inositol-free media (SC-inositol, Formedium). The list of the yeast strains used in this study is given in Table 1. *D. discoideum* lines were isogenic to the axenic strain AX2. Amoeba were cultivated at 22 °C in HL5 media, either in Petri dishes or in flasks at 120 rpm. Cells were diluted every 1–2 days to avoid confluence of dishes or when cell densities exceeded 5 × 10^6^ cells/ml.

**Table 1:** List of the yeast strains used in this study.

Name	Relevant genotype	Reference
DDY1810	*MATA leu2 ura3*-*52 trp1 prb1*-*1122 pep4*-*3 pre1*-*451*	
*kcs1Δ*	DDY1810 KCS1::LEU2	Onnebo and Saiardi, (2009)
*vip1Δ*	DDY1810 VIP1::KANMX4	Onnebo and Saiardi, (2009)
*kcs1Δvip1Δ*	DDY1810 KCS1::LEU2 VIP1::KANMX4	Onnebo and Saiardi, (2009)
*kcs1Δddp1Δ*	DDY1810 DDP1::LEU2 KCS1::KANMX4	This study
EY1109	MATa leu2::PHO84pr:GFP::LEU2 ADE2	Thomas and O'Shea, (2005)
EY1109 *pho81Δ*	MATa leu2::PHO84pr:GFP::LEU2 ADE2 PHO81::NatNT2	Desfougeres et al., (2016)
EY1109 *vip1Δ*	MATa leu2::PHO84pr:GFP::LEU2 ADE2 VIP1::NatNT2	This study

### 2.5 Quantification of the PHO pathway activation by fluorescence-activated cell sorting

Logarithmic growing yeast grown in Sc-Ura media carrying pADH-Ppip5k or empty vector were washed and shifted in media with or without phosphate 10 μM for 3 h. Before FACS measurement, 50 μl of the yeast culture was diluted into 950 μl of TBS and immediately analysed using an LSRII flow cytometer (BD Biosciences).

### 2.6 ^13^C-NMR analysis

*D. discoideum* AX2 and *ppip5k* were grown for 5–7 days in SIH media (Formedium) supplemented with ^13^C_6_-inositol (400 μM) synthesized as described (Harmel et al., 2019). Amoeba extracts were separated by PAGE to purify IP_7_ and IP_8_. In brief, whole-cell extracts from 350 ml labelled cultures were extracted with perchloric acid and run on single-lane 33% PAGE gels. Bands corresponding to each inositol pyrophosphate species were cut and elute over 24 h by rotation in alternating solutions of water and 1:1 water/methanol (Loss et al., 2011). The combined solutions were acidified with 0.1M perchloric acid and inositol pyrophosphate recovered by TiO_2_ purification as described (Wilson et al., 2015). The ^13^C,^1^H-NMR analysis was performed as previously described (Harmel et al., 2019) using a Bruker AVANCE III spectrometer.

### 2.7 Extraction and analysis of inositol polyphosphates form yeast

Logarithmic growing yeast were inoculated at OD_600_ = 0.01 in 5 ml of SC-Ura-inositol supplemented with 5 μCi/ml of [^3^H]-inositol. The yeast were grow for 16–20 h at 30 °C with shacking. Radiolabelled inositol phosphates were extracted and analysed by Sax-HPLC as described (Azevedo and Saiardi, 2006).

### 2.8 Extraction and PAGE analysis of inositol polyphosphates and polyP from amoeba

*D. discoideum* cells centrifuged (500 g, 4 min, 4 °C) and washed once with KK2 (20 mM potassium phosphate pH 6.8). Pellets were resuspended in 40–100 μl perchloric acid (1M), incubated in ice and vortexed for 10 s every 2 min for a total period of 10 min. The extracts were centrifuged at (18000 g, 5 min, 4 °C) and the supernatants were neutralised with 1M potassium carbonate supplemented with 3 mM EDTA at 4 °C for 2 h and subsequently centrifuged. For polyP analysis cell were extracted using acidic phenol procedure (Livermore et al., 2016). PAGE analysis over 33% acrylamide gel was performed as previously described (Losito et al., 2009). Briefly, gels were pre-run for 30 min before loading samples and running overnight at 700 V and 5 milliamps at 4 °C until the Orange G dye had run through 2/3 of the gel. Gels were stained by toluidine blue solution (20% methanol, 2% glycerol, 0.05% Toluidine Blue) at room temperature for 30 min with gentle agitation. Toluidine gels were destained twice in 20% methanol and scanned with an Epson Perfection 4990 Photo Scanner. Image quantification was carried using Image-J software package.

### 2.9 Generation of *D. discoideum* null strains

The *ip6k* and *ppip5k* strain were constructed using the TMO1 deletion vector (Muramoto et al., 2012). Regions of DNA flanking the gene of interest were amplified by PCR from AX2 genomic DNA using the oligo listed in Table 2. Knockout plasmids TMO1-IP6K-Bsr, TMO1-PPIP5K-Bsr, were generated by inserting these sequences into the plasmid TMO1, using NotI and *Eco*RI sites for the 5′ arm and the *Hin*dIII and *Kpn*I sites for the 3’ arm. The resultant plasmids were then digested using *Bss*HII and used to transform AX2 cells by electroporation using a Biorad Inc. genepulser and exposed to a single pulse of 0.65 kV at 25 μF. Amoebas were subjected to blastocidin selection before screening the transformants by PCR, southern and northern blot and biochemically by PAGE analysis. To generate the *ip6k-ppip5k* strain the Blastocidin Resistance gene was excised from the *ppip5k* strain using the pDex-Cre-NLS plasmid (dictybase stock centre (Faix et al., 2004), before knocking out the Ip6k gene using the strategy described above. Southern blotting and Northern blotting were performed using a standard procedure.

**Table 2:** Oligo use to generate the Ip6K and Ppip5k deletion constructs.

Gene	Primer	Primer sequence	Product length	Restriction site
Ip6k	5′ arm Forward	GCAGCGGCCGCCTCAATCCACCCACACTCAC	1050	NotI
	5′ arm Reverse	GCAGAATTCGTTGTTGTTGGGCTTCTTGG		*Eco*RI
	3′ arm Forward	GCGAAGCTTGCGGTAGTAAACCTCAACCTTC	835	*Hin*dIII
	3′ arm Reverse	GCAGGTACCCTCTTCACTGGTGACACCTATGC		*Kpn*I
Ppip5k	5′ arm Forward	GCAGCGGCCGCGTAGTCAGCAAATTTACCAC	1069	NotI
	5′ arm Reverse	GCAGAATTCCTATCTACAATTGGCCATTC		*Eco*RI
	3′ arm Forward	GCGAAGCTTGATATATTACGTGTTAATGG	832	*Hin*dIII
	3′ arm Reverse	GCAGGTACCTCAAATGGTCAAATTGCTGG		*Kpn*I

### 2.10 *D. discoideum* development

Amoebas were starved by transferring cells from rapidly dividing vegetative cultures onto KK2 2% agar plates. 1 × 10^7^ cells were plated on a 35 mm plate. Cells were allowed to develop at room temperature. Cells were collected after 1 h, ∼8 h when cells were beginning to aggregate, ∼16 h, when cells had coalesced to form slugs and after ∼24 h when fruiting bodies were fully formed. For development on filters, three Whatman® Grade 3 filter papers were layered and covered by a Whatman® Grade 50 quantitative filter paper (hardened low-ash). The filters were soaked in the specified buffer. Following the removal of excess liquid, cells were resuspended in 500 μl of buffer (KK2, TrisHCl 20 mM pH7.0 and HEPES 20 mM pH7.0) and allowed to flow into the filter by delivery with a pipette in an outward spiral movement. Cells were harvested with a plastic scraper. Fruiting body images were then taken on an Olympus camera mounted on a dissecting microscope.

### 2.11 *D. discoideum* CE-MS analysis

*D. discoideum* extract for CE-MS analysis were prepared from vegetative growing strains in HL5 medium. Cultures (20–30 ml of 1–3 X 10^6^ cell/ml) were spun at 500 g for 5 min; the cell pellet was washed in 1 ml KK2 and the inositol phosphate were extracted with 500 μl of perchloric acid 1M in the presence of 5 mM EDTA. The inositol phosphates in the perchloric acid extract were purified using TiO_2_ (Wilson et al., 2015) before subjecting them to CE-MS-Q-TOF analysis. The analysis was performed as previously described with internal standards (Qiu et al., 2020) using an Agilent 7100 capillary electrophoresis system coupled to a Q-TOF (6520, Agilent) mass spectrometer. Data were collected with Agilent OpenLAB CDS Chemstation 2.3.53 and Agilent MassHunter Workstation Acquisition for Q-TOF B.04.00.

## 3 Results and discussion

### 3.1 Identification of *D. discoideum* inositol phosphate kinases

The screening of *D. discoideum* genome revealed the presence of seven inositol phosphate kinases (Table 3). Out of these, only the Ip6k (gene name i6kA) has been characterized through the generation of the *ip6k* (*i6kA*) strain (Luo et al., 2003). Two other genes, IpkA and Ipmk, were used in overexpression studies but not biochemically characterized (Damstra-Oddy et al., 2021; King et al., 2010). As opposed to the four kinases found in yeast (Laha et al., 2021; Saiardi et al., 2018; Tsui and York, 2010), the seven *D. discoideum* kinases pointed towards a higher complexity of inositol phosphate synthesis in the amoeba, more similar to mammalian cells. Indeed, like the human genome, amoeba, possess Itpk1, the enzyme which drives the cytosolic route of IP_6_ synthesis (Desfougeres et al., 2019). The amoeba also possesses one IP5-2Kinase (yeast Ipk1 and mammalian IPPK) and one PPIP5K gene. The inositol kinase enzymology of amoeba is similar to the mammalian counterpart, therefore it is peculiar, as stated in the introduction, that amoeba and human inositol pyrophosphate species differ in their isomeric nature. We, decided to reinvestigate this issue by performing the new structural studies using the newly developed ^1^H,^13^C-NMR approach.

**Table 3:** Inositol phosphate kinases in *Dictyostelium discoideum*.

Gene ID	Dicty base name	Gene name	Inositol Kinase Family	Chr.	Yeast homol.	Mammalian homol.	Ref.
DDB_G0281737	*-*	*Ipmk*	IPK superfamily Pfam 03770	3	Arg82	IPMK	Damstra-Oddy et al., (2021)
DDB_G0278739	*I6kA*	*Ip6k*	IPK superfamily Pfam 03770	3	Kcs1	IP6K 1-3	Luo et al., (2003)
DDB_G0271760	*ipkA1*	*Ipka*	IPK superfamily Pfam 03770	2	–	–	King et al., (2010)
DDB_G0283863	*-*	*Ipkb*	IPK superfamily Pfam 03770	4	–	–	
DDB_G0269746	*Itpk1*	*Itpk1*	ITPK1 family Pfam 17927	1	–	ITPK1	
DDB_G0288351	*-*	*Ipk1*	IP5-2K family Pfam 06090	5	Ipk1	IPPK	
DDB_G0284617	*-*	*Ppip5k*	PPIP5K family Pfam 18086	4	Vip1	PPIP5K 1-2	

### 3.2 ^13^C-NMR characterization of *D. discoideum* IP_7_ and IP_8_

**Fig. 1:**
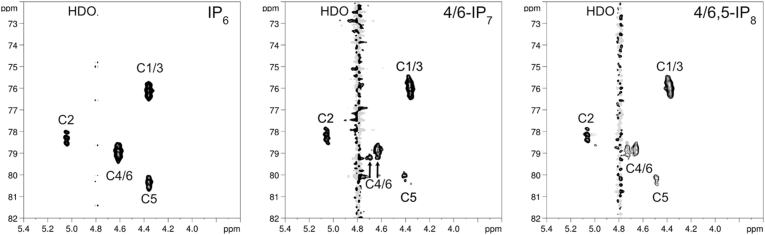
^13^C-NMR spectra of IP_6_, IP_7_ and IP_8_ extracted from *D. discoideum* AX2. Inositol pyrophosphates (extracted and TiO_2_-purified from *D. discoideum* grown in presence of ^13^C6-inositol) were analysed using a Bruker AVANCE III spectrometer operating at 600 MHz 600 MHz for proton, and at 151 MHz for carbon. Two-dimensional ^1^H,^13^C NMR spectrum for IP_6_ (left), IP_7_ (centre) and InsP_8_ (right) reveals the presence of 4/6-IP_7_ and 4/6,5-IP_8_ in wild type amoeba. The arrows indicate the spitted 4/6 carbon signal in 4/6-IP_7_. The positions of the carbon atoms and the solvent signal of deuterium water (HDO) are indicated.

Previous NMR studies of IP_7_ and IP_8_ purified from amoeba were performed using two-dimensional ^1^H,^31^P-NMR (Laussmann et al., 1996). This approach has limited sensitivity. Conversely, the newly developed ^1^H,^13^C-NMR offers higher sensitivity since the chemical shift dispersion of ^13^C is superior to ^31^P, and the magnetization transfer *via* ^1^J(^1^H,^13^C) one-bond couplings is more efficient (Harmel et al., 2019). We fed wild type AX2 amoeba with ^13^C_6_-inositol and after extracting and purifying the inositol pyrophosphates using TiO_2_, we analysed them using ^1^H,^13^C-NMR spectroscopy. The 2-dimensional inverse H,C correlation spectra (Fig. 1) confirm previous studies. The IP_7_ spectra reveal the carbon 4/6 split signal, while the IP_8_ spectra additionally reveal the shift towards the left of the signal of carbon 5. These are the typical signatures of pyrophosphate moieties at these two carbons (Harmel et al., 2019). Therefore, the inositol pyrophosphate isomers present in the social amoeba are indeed the 4/6-IP_7_ and the 4/6,5-IP_8_ forms. Of note, neither the myo-inositol 4/6 carbon positions nor the 1/3 carbon positions can be distinguished by NMR as they are enantiotopic. The different inositol pyrophosphate isomers present in amoeba and mammals, despite similar enzymology, suggests that the IP6K or the PPIP5K enzyme could pyro-phosphorylate different inositol ring positions depending on the species analysed.

### 3.3 *D. discoideum* Ppip5K rescues yeast vip1Δ phenotypes

**Fig. 2:**
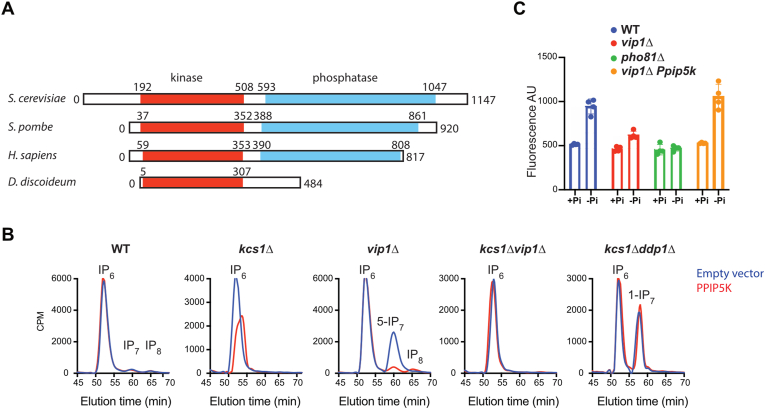
*D. discoideum* Ppip5k structure and its ability to rescue yeast *vip1Δ* phenotypes. The schematic representation of Ppip5k structural organization (A) from different organisms illustrates the absence of the phosphatase domain in *D. discoideum* protein. Sax-HPLC analysis of ^3^H-inositol labelled yeast expressing amoeba Ppip5K (B) reveals the ability of this enzyme to revert the biochemical phenotype of the *vip1Δ strain, i.e.* an increase in 5-IP_7_. The activation of the PHO pathway under phosphate starvation was monitored by FACS analysis (C). In the indicated strains, GFP is under the control of the promoter of the high affinity phosphate transporter Pho84 and is thus a readout for the PHO pathway activation. The different yeast carrying empty vector or pADH-Ppip5k were washed and shifted in media with (+Pi) or without (-Pi) phosphate for 3 h. The defect in the response observed in the *vip1*Δ is fully rescued upon expression of Ppip5k. The *pho81Δ* strain, which constitutively represses the PHO pathway, is used as a control. The results are from four independent experiments.

To gain further insight into *D. discoideum* inositol pyrophosphate metabolism, we focused our attention on Ppip5k. The Ppip5k (DDB_G0284617) homologue in *D. discoideum* encodes a 56 kDa protein, compared to the 130 kDa yeast protein and ∼150 kDa in mammalian cells (Fig. 2A). In both yeast and mammals, the PPIP5K encodes a protein containing both a kinase domain and a phosphatase domain (Dollins et al., 2020; Pascual-Ortiz et al., 2018). Interestingly, the *D. discoideum* gene encodes a much smaller enzyme, which completely lacks the phosphatase domain. The absence of this phosphatase domain in the amoeba might abolish the futile cycle proposed for this type of kinase (Randall et al., 2020).

The *D. discoideum* proteome has evolved to encode peptides with long poly-glutamine or poly-asparagine tracts (Santarriaga et al., 2015). The Ppip5k coding sequence possesses two long poly-asparagine stretches that have prevented us obtaining recombinant Ppip5k from bacterial expression systems. Similarly, *D. discoideum* Ip6k (DDB_G0278739) contains six poly-asparagine and one poly-glutamine repeats, which have also prevented us obtaining recombinant protein. Therefore, to test the amoeba Ppip5k activity, we cloned the gene into a yeast expression vector and transformed it into an array of *S. cerevisiae* mutants (Fig. 2B). The amoeba Ppip5k is able to completely reverse the increase in 5-IP_7_, observable in *vip1*Δ yeast. Ppip5k do not appear to use as substrate the 1-IP7 that accumulate in *kcs1*Δ*ddp1*Δ or the IP_6_ present in *kcs1*Δ or *kcs1*Δ*vip1*Δ strains.

To verify if the biochemical phenotype generates a product functionally equivalent to the Vip1 generated IP_8_, we investigated if amoeba Ppip5k rescues the *vip1*Δ PHO response defect (Choi et al., 2017). In low phosphate conditions, a set of genes, named the *PHO* genes, are up-regulated. The expression of these genes is repressed in phosphate-rich conditions. One such gene is *PHO8*4 that encodes a high affinity phosphate transporter. The induction of the *PHO* genes expression can be monitored by recording the expression of a reporter-protein cloned behind the *PHO* gene promoter. We took advantage of the strain (EY1109) developed by Thomas and O'Shea that expresses GFP under the control of the PHO84 promoter (Thomas and O'Shea, 2005). In this background, the deletion of *Vip1* leads to a repression of the *PHO* genes expression, as previously demonstrated (Choi et al., 2017). This is similar to what is observed when *PHO81* is deleted (Desfougeres et al., 2016). Expression of the amoeba Ppip5k in the *vip1Δ* strain fully rescues the expression of the reporter (Fig. 2C) indicating that, *in vivo*, the product of the enzymatic reaction catalysed by Ppip5k is functionally equivalent to the Vip1 product. The rescue of both biochemical and physiological *vip1Δ* phenotypes demonstrates that *D. discoideum* Ppip5K is a genuine PPIP5K enzyme able to phosphorylate 5-IP_7_ to, likely, 1,5-IP_8_.

### 3.4 Generation of the *D. discoideum* ppip5k strain

**Fig. 3:**
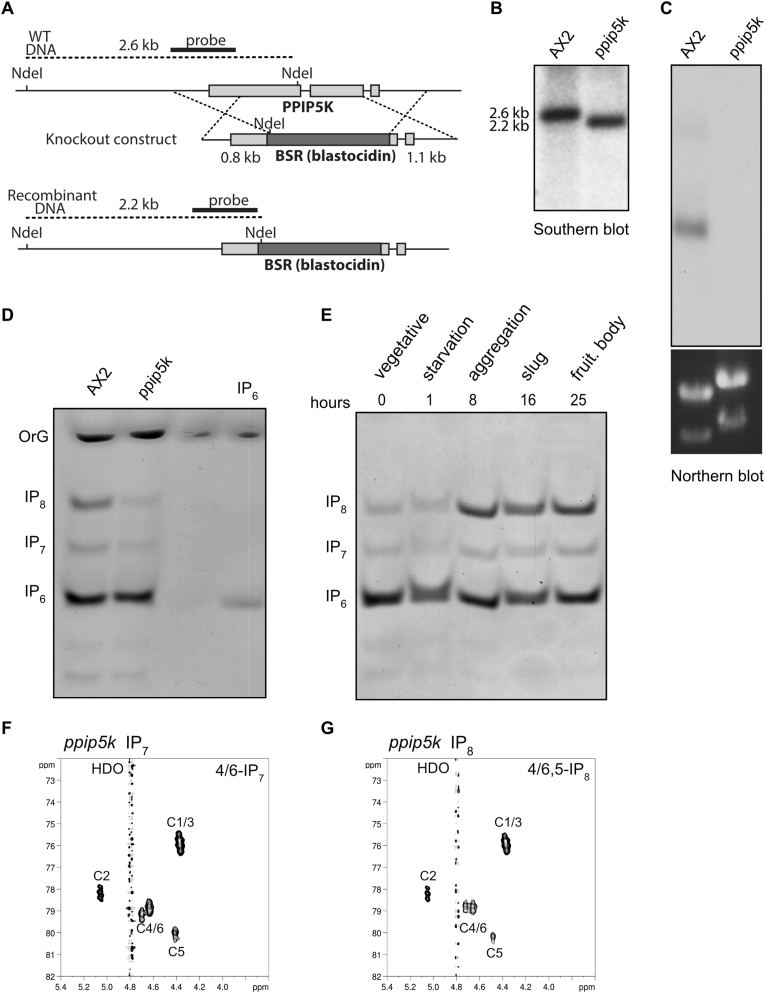
Generation of *D. discoideum ppip5k* strain. The homologous recombination strategy to generate *ppip5k* amoeba (A) highlights the screening approach and probe location. Southern blot analysis (B) of AX2 and *ppip5k* amoeba is consistent with the strategy design prediction. Norther blot analysis (C) reveals the absence of any Ppip5k transcript in the *ppip5k* amoeba. Ethidium bromide staining (bottom panel) of the ribosomal RNA confirms equal loading. Neutralised acidic extracts from AX2 and *ppip5k-* (5 × 10^6^ cells) were resolved on 33% PAGE and the inositol phosphates were visualised by toluidine blue staining (D). This analysis reveals a substantial decrease in the level of IP_7_ and especially IP_8_, in the *ppip5k* strain. The PAGE is a representative result of an experiment repeated 4 or more times. The recorded accumulation of IP_8_ during development (Pisani et al., 2014) is not altered in the *ppip5k* amoeba (E) as revealed by PAGE analysis of neutralised cell extracts collected at different developmental stages. The experiment was repeated twice giving identical results. Two-dimensional ^1^H,^13^C NMR spectrum of *ppip5k* extracted IP_7_ (F) and IP_8_ (G) reveals the presence of 4/6-IP_7_ and 4/6,5-IP_8_ like in wild type amoeba. The positions of the carbon atoms and the solvent signal of deuterium water (HDO) are indicated.

Since NMR studies indicates that *D. discoideum* do not possess 1,5-IP_8_ ((Laussmann et al., 1996) Fig. 1), we decided to knockout Ppip5k to characterise the effect of the absence of this kinase on the amoeba inositol pyrophosphate metabolism. A homologous recombination approach was used to generate amoeba knockout. This approach involved cloning two regions flanking the target gene and inserting them on either side of a Blastocidin resistance marker gene. The strategy for deletion of the Ppip5k involved cloning 1.1 kb of genomic sequence overlapping slightly with the 5′ region of the gene and 0.8 kb 3’ of the gene (Fig. 3A). The generated *ppip5k* strains were confirmed by Southern blot (Fig. 3B); while Northern blot analysis (Fig. 3C) confirms the loss of the Ppip5K transcript in the *ppip5k* strain.

The analysis by PAGE of the *ppip5k* inositol pyrophosphates profile reveals a 49.6 ± 7.8% (n = 4) reduction in IP8 levels while a 14.3 ± 6.7% (n = 4) reduction in the level of IP_7_ is recorded (Fig. 3D). This biochemical defect is remarkably different from the one reported for yeast *vip1*Δ in which a substantial increase in IP_7_ is observed (Onnebo and Saiardi, 2009). The decrease of both IP_7_ and IP_8_ observed in *ppip5k* amoeba grown in rich HL5 medium, prompted us to verify if the reported increase on IP_8_ level during *D. discoideum* development (Laussmann et al., 2000; Pisani et al., 2014) is under Ppip5k control. The developmental analysis of inositol pyrophosphate profile in *ppip5k* amoeba reveals a consistent accumulation of IP_8_ during the late stage of development. In conclusion, *ppip5K* regulate inositol pyrophosphate metabolism in the vegetative stage but not its modulation during amoeba development (Fig. 3E).

We next assessed the isomeric nature of IP_7_ and IP_8_ in the *ppip5k* strain using ^13^C-NMR. After feeding *ppip5k* amoeba with ^13^C_6_-inositol IP_7_ and IP_8_ were extracted and subjected to ^13^C-NMR analysis (Fig. 3F and G). The *ppip5k*-purified IP_7_ and IP_8_ spectra show the characteristic signature of pyrophosphate moiety at position 4/6 and 5 carbons. Like the wild type AX2 amoeba, the *ppip5k* strain possesses the 4/6-IP_7_ and 4/6,5-IP_8_ isomers. This is not surprising since PPIP5K are kinases thought to phosphorylate position one of the inositol ring (Lin et al., 2009; Wang et al., 2012); furthermore our *vip1*Δ rescue experiments (Fig. 2B and C) are also indicative of this specificity. While ^13^C-NMR sensitivity might fail to detect minor species of IP_7_ and of IP_8_ species, our theoretical consideration and our analysis suggest that amoeba Ppip5k, while not participating directly in the synthesis of the abundant 4/6,5-IP_8_, is nevertheless able to regulate its cellular levels.

### 3.5 Generation of the *D. discoideum* ip6k and ip6k-ppip5k strain

**Fig. 4:**
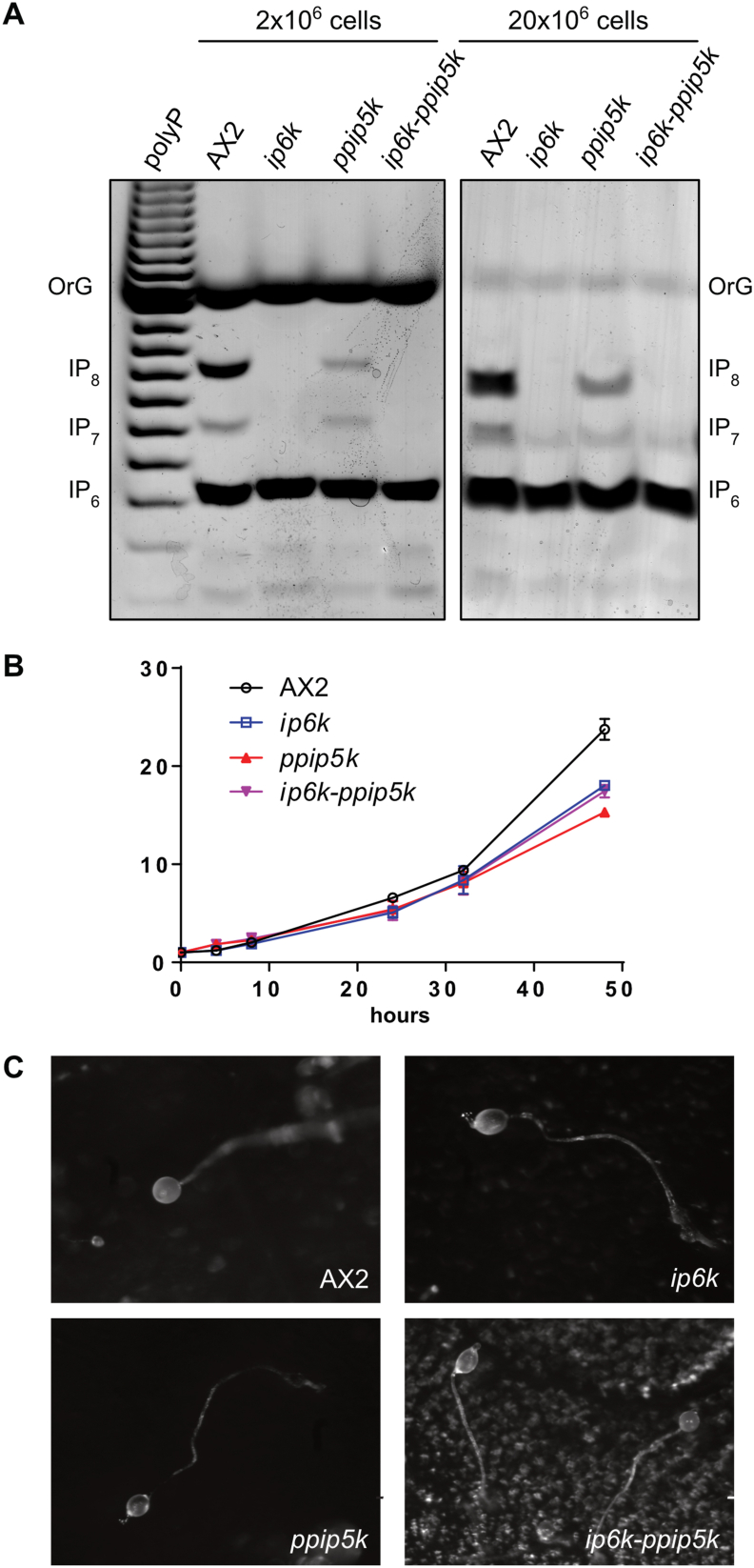
Comparative biochemical, growth and developmental analysis of *D. discoideum* wild type and knockout strains. Inositol pyrophosphates extracted from 1 × 10^6^ (left) or 20 × 10^6^ (right) AX2 and mutants *ip6k*, *ppip5k* and *ip6k-ppip5*^*-*^ amoeba were resolved on 33% PAGE and visualised by staining with toluidine blue (A). Inorganic polyphosphate polyP is used to orientate the gel, OrangeG (OrG) is used as migrating dye. A reduction but not ablation of both IP_8_ and IP_7_ is noticeable in *ppip5k* strain. Neither IP_7_ nor IP_8_ is detectable in extracts from two million cells of either *ip6k* or *ip6k-ppip5k* (left panel). However, residual levels of IP_7_ are detectable in extracts from 20 millions of cells of both *ip6k* and *ip6k-ppip5k* (right panel). The gel is representative of at least three independent experiments. To investigate the effect of the altered inositol pyrophosphate metabolism on general fitness, the growth of AX2 was compared to *ip6k*, *ppip5k* and *ip6k-ppip5k* mutants (B). WT, *ip6k, ppip5k* and *ip6k-ppip5k* were grown for 48 h in HL5 media starting at a density of 1 × 10^5^ cells per ml The figure shows averages ± SD from three independent experiments. All three mutants strain displayed very slight growth defects not reaching statistical significance. Developmental analysis (C) performed under standard KK2-agar conditions shows no obvious developmental phenotype as revealed by the photos of the fruiting body. The result is representative of an experiment repeated at least three times.

The inability to produce recombinant Ip6k and Ppip5k proteins to assess their biochemistry *in vitro*, prompted us to develop the full array of knockout strains to perform *in vivo* analyses. We re-generate the *ip6k* strain (see material and methods) isogenic to our AX2 background. Identically to the previously generated ip6k mutant, the new strain has no detectable level of IP_8_ and an almost completely depleted level of IP_7_ (Fig. 4A). Double mutants, in which both *ppip5k* and *ip6k* genes were disrupted, were generated starting from the *ppip5k* strain in which the blastocidin resistance gene (BSR) was excised by overexpressing a recombinant Cre (Faix et al., 2004). The Ip6k gene was then disrupted to generate the *ip6k-ppip5k* strain (Fig. 4A). PAGE analysis of the *ip6k-ppip5k* reveals an inositol pyrophosphate profile similar to *ip6k* amoeba. Both the *ip6k* and the *ip6k-ppip5k* strains possess residual amounts of IP_7_ detectable by PAGE when extracts from 20 million cells were loaded on gel (Fig. 4A right panel), indicating the presence of an additional enzyme able to synthesize inositol pyrophosphates.

**Fig. 5:**
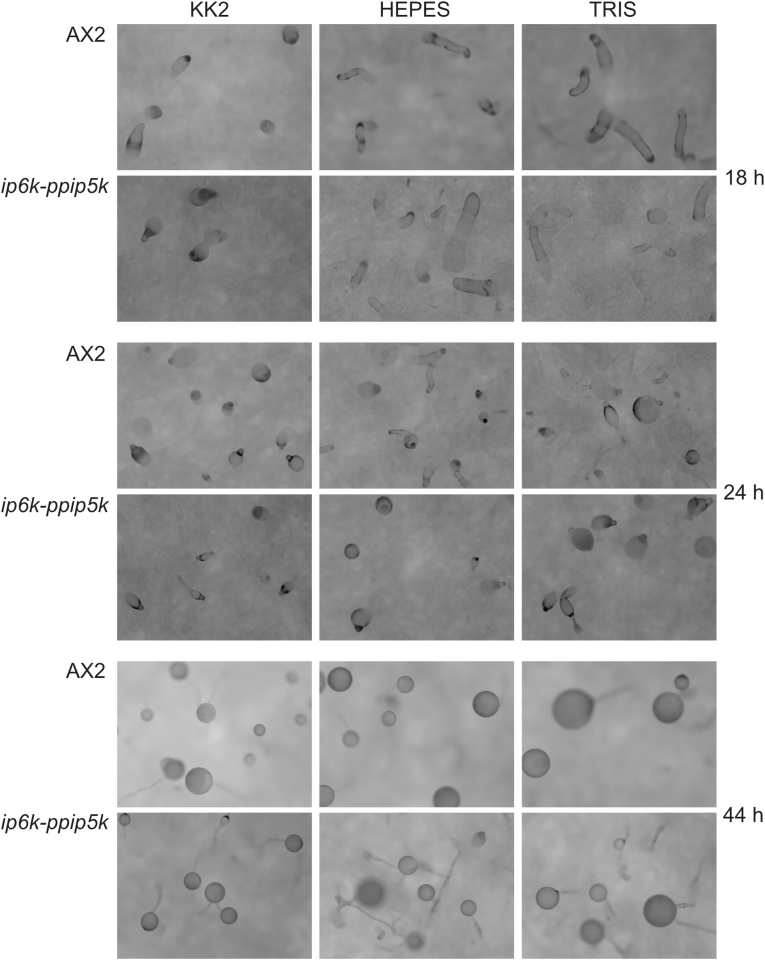
Developmental progression of wild type AX2 and the double knockout *ip6k-ppip5k* strain. To assess if phosphate affects the developmental process of the *ip6k-ppip5k* strain, the AX2 and *ip6k-ppip5k* were developed on buffered cellulose filters. The buffers used were KK2 (potassium phosphate buffer), the phosphate-free HEPES buffer, and TRIS buffer. While developmental timings were inconstant between experiments, due to the variable amount of liquid in the imbedded filter, no consistent phenotype was detected.

To verify the effect on the amoeba general fitness of the deletion of the known inositol pyrophosphate synthesizing enzymes, we characterized the growth rate of AX2, *ip6k, ppip5k*, and *ip6k-ppip5k* amoebas (Fig. 4B). We did not observe major growth defects when the null strains were grown in rich HL5 synthetic media. Although we could observe the tendency for the mutant strains to grow slowly, this difference does not reach statistical significance. We next assessed the ability of the mutants to undergo starvation-induced development. All strains succeeded to follow development under standard KK2 agar conditions and form fruiting bodies (Fig. 4C). To detect any developmental phenotype that may have gone unnoticed in a phosphate rich buffer as KK2 (20 mM potassium phosphate buffer pH 6.8), the process of development of the double mutant *ip6k-ppip5k* was examined under complete phosphate starvation using TrisHCl or HEPES as buffer on cellulose filters. Despite variable differences in timing, not attributable to differing buffer conditions, *ip6k-ppip5k* completed development, culminating in the formation of fruiting bodies slightly smaller than the AX2 strain (Fig. 5).

### 3.6 CE-MS analysis of *D. discoideum* inositol pyrophosphate metabolism

To better elucidate the inositol pyrophosphate metabolism in the mutant strains, we next performed Capillary Electrophoresis Mass Spectrometry (CE-MS) analysis (Qiu et al., 2020). This sensitive analytical technique resolves with unprecedented resolution the different isomers of IP_7_ and IP_8_. CE-MS studies complement ^13^C-NMR analysis, which offers unique structural information, but lacks the degree of sensitivity of mass spectrometry detection.

**Fig. 6:**
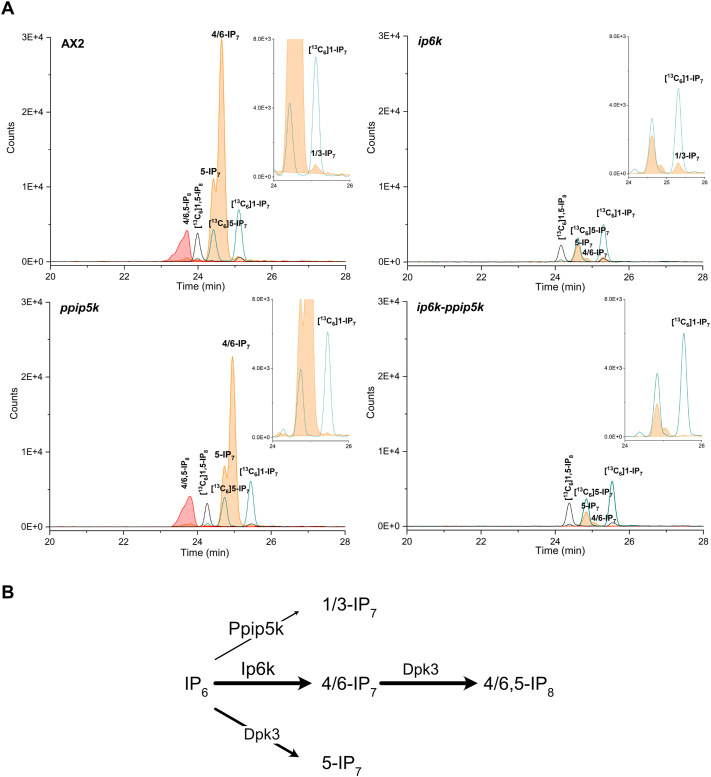
CE-MS analysis of *D. discoideum* of AX2 and *ip6k* and *ip6-ppip5k* strains extracts. Qualitative CE-MS separation of TiO_2_-purified *D. discoideum* extracts (A). Empty peak area indicates the migration of the indicated ^13^C_6_-inositol standard while the filled peak area represents the elution of the amoeba extracted IP_7s_ (orange) and IP_8_ (red). Enlarged inserts for the IP_7_ region are presented to highlight the minor species of 1-IP_7_ and 5-IP_7_. This analysis reveals the absence of 1-IP_7_ in *ppip5*^*-*^ and of 4/6-IP_7_ in *ip6k* stains and of both in *ip6k-ppip5k*^*-*^. From this, we could deduce the inositol pyrophosphate pathway presented in (B) where Dpk3 stand for Diphosphate kinase number 3. This analysis was repeated three times giving identical results.

The qualitative analysis of AX2 amoeba reveals 4/6-IP_7_ and 4/6,5-IP_8_ to be the major inositol pyrophosphates species, confirming the ^13^C-NMR studies (Fig. 6). However, two additional, IP_7_ isomers could be identified; 5-IP_7_ constituting about 20% of the entire IP_7_ pools and 1-IP_7_ representing roughly <5% of the entire IP_7_ pools. As expected, the analysis of *ppip5k* strain reveals the absence of the minor 1/3-IP_7_ species demonstrating that the *D. discoideum* Ppip5k likely phosphorylates the 1 position similarly to the mammalian counterpart (Wang et al., 2012) and as our yeast rescues experiment suggested (Fig. 2B and C). The Ppip5k synthesized 1/3-IP_7_, while not participating directly in the synthesis of the 4/6,5-IP_8_, still regulates its cellular level (Fig. 3, Fig. 4A).

Surprisingly, the analysis of *ip6k* mutant reveals the disappearance of the major IP_7_ isomer the 4/6-IP_7_ species (Fig. 6A). Thus, to the contrary of the mammalian IP6Ks *D. discoideum* homologous enzyme pyrophosphorylate position 4/6 of the inositol ring generating 4/6-IP_7_. In light of these observations, in the *ip6k-ppip5k* amoeba, only the 5-IP_7_ isomer could be detected. Therefore, *D. discoideum* must possess an additional kinase that we named Diphospho kinase 3 (Dpk3) responsible for 5-IP_7_ synthesis and that together with the Ip6k would generate 4/6,5-IP_8_. Fig. 6B summarises *D. discoideum* inositol pyrophosphate metabolism revealed by these analyses where Dpk3 represents an as-yet uncharacterised kinase capable of producing inositol pyrophosphates. The presence of three IP_7_ isomers suggests the possibility for amoeba to synthesize three IP_8_ species however only 4/6,5-IP_8_ is detectable in our current CE-MS-qTOF experimental setup.

### 3.7 polyP metabolism is not affected by *D. discoideum* Ip6k or Ppip5k

Based on our understanding of the link between inositol pyrophosphates and phosphate metabolism in yeast, the altered inositol pyrophosphates present in *ip6k, ppip5k,* and *ip6k-ppip5k* could influences directly or indirectly phosphate homeostasis in *D. discoidem*. The social amoeba possess sub-millimolar concentration of the phosphate-rich IP_6_, IP_7_, and IP_8_ thus changing their concentration influences a large pool of cellular phosphate. Alternatively, inositol pyrophosphate could influence phosphate availability by regulating polyP metabolism, particularly as the primary function of polyP is to buffer cellular free phosphate concentration. We previously demonstrated that in *S. cerevisiae* polyP synthesis is under Kcs1 (the IP6K) control (Lonetti et al., 2011), while in *S. pombe* it is Asp1/Vip1 (the PPIP5K) that regulates polyP metabolism (Pascual-Ortiz et al., 2021). Therefore, in yeast, there is a clear link between inositol pyrophosphate and polyP cellular level even if the precise inositol phosphate kinase regulating polyP metabolism differs between yeast species.

**Fig. 7:**
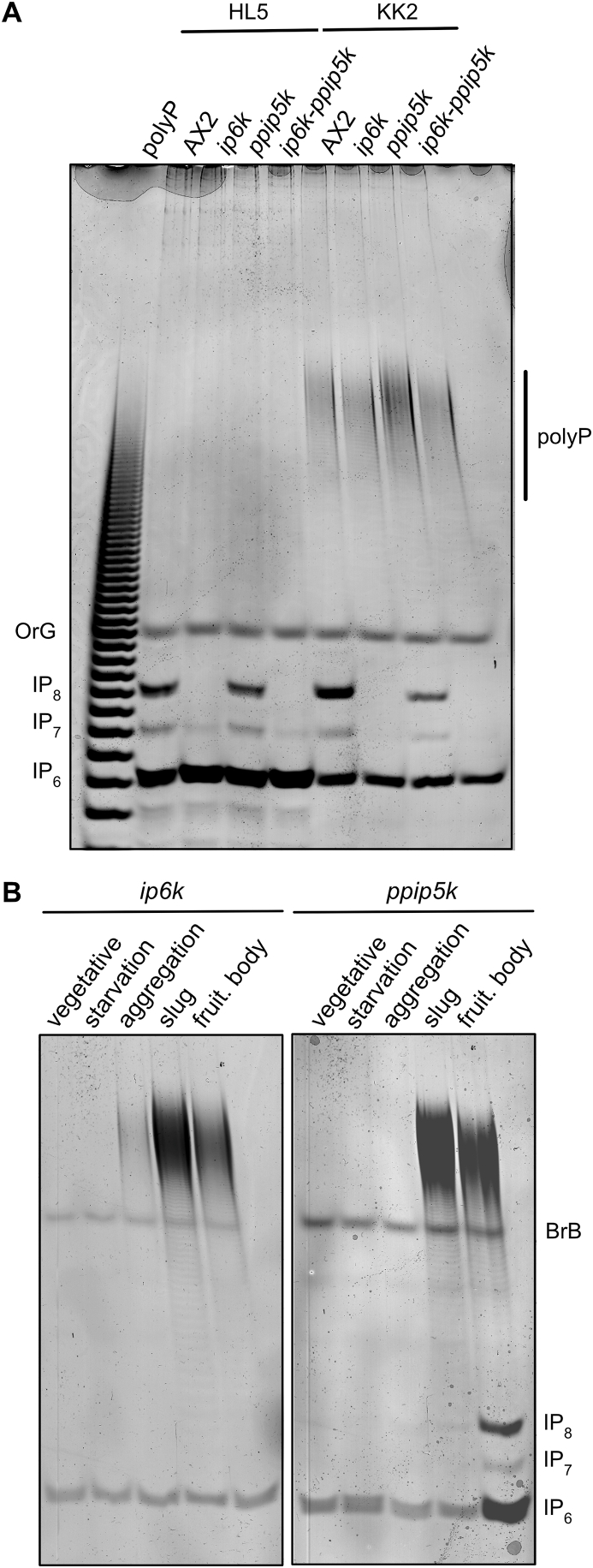
*D. discoideum* polyP analysis of vegetative and development states of the inositol phosphate kinase mutants. Wild type AX2 and *ip6k* and *ip6k-ppip5*^*-*^ strains growing exponentially in rich medium HL5 were collected (20 × 10^6^ cells) or plated on KK2 agar plates for 16 h to stimulate developmentally induced polyP synthesis. The plates were scraped to recover *D. discoideum* and a neutralised acidic extract of the samples in vegetative stage (HL5) or after starvation (KK2) was prepared before loading on a 33% PAGE gel. The gels were stained with Toluidine blue to visualise inositol phosphate and polyP. While in this experimental set up polyP could not be detected in vegetative amoeba, polyP induction during development is clearly evident in both the wild type or mutant stains. OrangeG (OrG) is used as migrating dye. Developmental accumulation of polyP (B) is present in both *ip6k* (left panel) and in *ppip5k* (right panel) strains. Bromophenol Blue (BrB) is used as migrating dye. The figure is representative of experiments repeated at least three times.

The *D. discoideum ip6k, ppip5k,* and *ip6k-ppip5k* strains offer the opportunity to verify in an organism belonging to a different taxon if the synthesis of polyP is under control of Ip6K, of Ppip5k, of both or neither of the two enzymes. We extracted polyP using acidic phenol procedure from fast-dividing AX2, *ip6k, ppip5k*, and *ip6k-ppip5k* amoeba grown on rich HL5 medium and from KK2 agar plates for 16 h, a condition we previously demonstrated to induce polyP synthesis (Livermore et al., 2016). PAGE analysis of the extracted polyP revealed that while polyP is undetectable in this experimental setup from amoeba grown in HL5 medium, polyP under starvation conditions is detected in all four *D. discoideum* strains (Fig. 7A). We next followed the developmental synthesis and accumulation of polyP (Livermore et al., 2016). The *ip6k* and *ppip5k* amoebae were transferred on KK2 agar and cells were collected at different time points corresponding to the diverse developmental stages. PAGE analysis of phenol extract from *ip6k* and *ppip5k* amoebae revealed the dramatic accumulation of polyP during development as reported previously (Livermore et al., 2016). Therefore, in the social amoeba nether, the Ip6K or the Ppip5k are able to control polyP metabolism.

## 4 Conclusion

Studying the inositol pyrophosphate metabolism in *ip6k, ppip6k* and *ip6k-ppip5k* amoeba revealed interesting features. The amoeba Ip6k synthesizes 4/6-IP_7_ instead of the 5-IP_7_ isomer synthesized by its mammalian counterpart. Therefore, the definition of the inositol pyrophosphate species present in one specific organism cannot be extrapolated by sequence homology, but must be tested experimentally.

Conversely, the amoeba Ppip5k similarly to its mammalian counterpart, does pyro-phosphorylate position 1/3 of the inositol ring producing 1/3-IP_7_, and therefore could not participate directly to the synthesis of the abundant 4/6,5-IP_8_ isomer. The 1/3-IP_7_ is by far the minor species of the three IP_7_ isomer found in amoeba, but it does indirectly regulate IP_8_ synthesis since *pppip5k* possess a 50% decrease in 4/6,5-IP_8_ level. These results indicate the existence of a third kinase, likely regulated by Ppip5k or its product, able to pyro-phosphorylate position five, synthesizing 5-IP_7_ and 4/6,5-IP_8_. The recent discovery that *Arabidopsis thaliana* possesses three isomers of IP_7_ and like in *D. discoideum* the most abundant is the 4/6-IP_7_ species (Riemer et al., 2021), suggests the amoeba inositol pyrophosphates metabolism is conserved across many species.

Our work also reveals that in amoeba neither the Ip6k or the Ppip6k are involved in regulating polyP metabolism. This should not come as a surprise, since the synthesis of polyP in amoeba and yeast occurs using different enzymology. While yeast Vtc4 possesses an SPX domain that could be regulated by inositol pyrophosphates, the amoeba Ppk1 does not. Our work highlights how incorrect it is to extrapolate polyP yeast discoveries to other species when the mechanism of polyP synthesis is different as in amoeba or unknown as in mammals.

Surprisingly, *ip6k, ppip6k* and *ip6k-ppip5k* amoebas do not show major growth or developmental defects. We could not exclude that the minor species of IP_7_ present in *ip6k* and the 5-IP_7_ present in *ip6k-ppip5k* is sufficient to play signalling roles preventing the manifestation of inositol pyrophosphate-specific phenotypes. For this reason, it is imperative to identify the *D. discoideum* enzyme responsible to pyro-phosphorylate position five of the inositol ring Dpk3 (Fig. 6B) and thus responsible for the synthesis of the 5-IP_7_ present in *ip6k-ppip5k* and for the synthesis of the abundant 4/6,5-IP_8_ present in wild type amoeba. After identifying this additional kinase, the generation of the triple mutant strain might reveal the amoeba phenotypes associated with the absence of inositol pyrophosphates.

## Credit author statement

Yann Desfougères: Investigation, Visualization, Writing- Reviewing and Editing. Paloma Portela-Torres: Investigation, Visualization, Writing – review & editing. Danye Qiu: Investigation, Visualization, Writing – review & editing. Thomas M Livermore: Investigation, Writing – review & editing. Robert K Harmel: Investigation, Visualization, Writing – review & editing. Filipy Borghi: Writing – review & editing. Henning J. Jessen: Supervision, Formal analysis, Funding acquisition, Dorothea Fiedler: Supervision, Formal analysis, Funding acquisition, Adolfo Saiardi; Conceptualization, Methodology, Supervision, Formal analysis, Funding acquisition, Writing – original draft, Writing – review & editing.

## Funding

This work was supported by the Medical Research Council (MRC) grant MR/T028904/1, and by the European Union's Horizon 2020 research and innovation program under the 10.13039/100010665Marie Skłodowska-Curie Grant agreement PHEMDD 752903. This study was supported by the 10.13039/501100001659Deutsche Forschungsgemeinschaft (DFG) under Germany's Excellence Strategy (CIBBS, EXC-2189, Project ID 390939984 to HJJ). D.Q. gratefully acknowledges financial support from the Brigitte-Schlieben-Lange-Programm. R.K.H. gratefully acknowledges funding from the 10.13039/501100001664Leibniz-Gemeinschaft (SAW-2017-FMP-1).

## Declaration of competing interest

The authors declare none conflict of interest. The funding bodies do not have any role in the study design, and in data collection and analysis.

## Abbreviations

IP6: inositol hexakisphosphate, phytic acid
IP7: diphosphinositol pentakisphosphate
IP8: bis-diphosphinositol tetrakisphosphate
IP6K: inositol hexakisphosphate kinase
PPIP5K: diphosphoinositol pentakisphosphate kinase
Vtc4: vacuolar transporter chaperone 4
PPK1: polyphosphate Kinase 1
polyP: inorganic polyphosphate
SPX: protein domain named after SYG1/Pho81/XPR1
AX2: axenic strains 2
NMR: nuclear magnetic resonance
CE-MS: capillary electrophoresis mass spectrometry
qTOF: quadrupole time of flight
BSR: blastocidin resistance gene
PAGE: poly acrylamide gel electrophoresis
OrG: Orange G
BrB: bromophenol blue
DpK3: diphospho kinase 3
